# Concomitant ^177^Lu-DOTATATE and capecitabine therapy in malignant paragangliomas

**DOI:** 10.1186/s13550-019-0484-y

**Published:** 2019-02-06

**Authors:** Madhav Prasad Yadav, Sanjana Ballal, Chandrasekhar Bal

**Affiliations:** Department of Nuclear Medicine, All India Institute of Medical Sciences, Ansari Nagar, New Delhi, 110029 India

**Keywords:** Paraganglioma, ^177^Lu-DOTATATE, Capecitabine

## Abstract

**Background:**

The role of concomitant peptide receptor radionuclide therapy (PRRT) and capecitabine therapy has shown benefit in gastroenteropancreatic neuroendocrine tumors. However, data reporting its role in paraganglioma (PGL) patients is lacking. The aim of this study was to evaluate the role of combined capecitabine and ^177^Lu-DOTATATE in malignant PGL patients.

**Methods:**

In this retrospective, single-institutional, single-arm, observational study, data of consecutive advanced stage PGL patients treated with concomitant ^177^Lu-DOTATATE-capecitabine therapy, between July 2009 and March 2017, were collected and analyzed.

**Results:**

Twenty-five PGL patients received an average dose of 22.86 ± 9.54 (14.43–50) GBq ^177^Lu-DOTATATE and 1250 mg/m^2^ capecitabine from days 0 to 14, commencing on the morning of PRRT. The median overall survival (OS) was not attained in this patient cohort; however, the median PFS was 32 months. Morphological response according to RECIST 1.1 criteria was achieved in 28% (7/25) patients. Biochemical response with > 50% reduction in chromogranin A levels was observed in 28% of the patients.

**Conclusions:**

Our data confirm that ^177^Lu-DOTATATE-capecitabine therapy is effective in achieving an objective response in 28% and symptomatic response in 43% patients. In comparison to published PRRT monotherapy outcomes in PGL, we did not observe any great advantage of concomitant therapy; however, it could be due to under-powered study. We recommend a large randomized trial to prove or disprove the utility of capecitabine as a radiosensitizer for PRRT in PGL patients.

## Background

Paragangliomas (PGLs) are catecholamine-secreting tumors with neuroendocrine (NET) characterization that arise from neural crest cells of extra-adrenal autonomic ganglia. PGLs occur in both the sympathetic and parasympathetic nervous system. Normally, parasympathetic PGLs are non-secretory. The sympathetic PGLs secrete catecholamines and produce symptoms such as secondary hypertension, headache, sweating, palpitations, and anxiety [1, 2]. Malignancy of pheochromocytoma (PCC)/PGL, considered as intra-adrenal PGLs, ranges from 3 to 36% with lower rates in PCC compared to PGL due to either metastatic spread of the disease or recurrence after treatment [3]. The best treatment is complete surgical resection but is challenging in the case of local invasion and in patients where there is a systemic spread of disease. Radiation therapy > 40 Gy provides local tumor control and relief of symptoms [4–6]. Sunitinib, a tyrosine kinase inhibitor, has shown clinical benefit in 47% of patients with progressive PCC/PGL, demonstrating however a poor median PFS of 4.1 months [7]. Considering the limited curative options for metastasized PGL patients, the benefits and side effects of therapeutic interventions should be carefully weighed. Systemic therapeutic options like radionuclide therapy initially included ^131^I-metaiodobenzylguanidine (MIBG) therapy for tumors demonstrating avid ^123^I/^131^I-MIBG uptake on imaging. Studies have documented partial response, disease stabilization, and control of symptoms by improvement in the performance status with ^131^I-MIBG therapy [8–12]. However, the therapy is limited in patients with negative ^131^I-MIBG uptake on imaging. Moreover, the prolonged hospital admission in the isolation room and significant hematological toxicity of ^131^I-MIBG therapy are limiting factors [10].

PGL tumors have overexpression of somatostatin receptors (SSTR) on the cell surface which can be exploited as a novel molecular target. ^177^Lu-DOTATATE is eventually replacing ^131^I MIBG therapy and is considered as a current therapeutic option for PGL. Till date, the high therapeutic effectiveness of PRRT in gastroenteropancreatic neuroendocrine tumors (GEP NETs) is well established [13–15]. However, literature regarding the therapeutic efficacy of ^177^Lu-DOTATATE therapy in patients with PGL is limited to only few case series and case reports with the largest series published by Forrer et al. [16].

Capecitabine is an oral 5-FU prodrug and mimics the pharmacokinetics of 5-FU. Its radiosensitizing effects are a result of its ability to prevent DNA synthesis through inhibition of thymidylate synthase. In a two-armed study by our group on ^177^Lu-DOTATATE therapy involving 167 patients who had GEP NETs, combined ^177^Lu-DOTATATE-capecitabine therapy was superior to conventional ^177^Lu-DOTATATE therapy alone [17]. Till date, there is only one such study reported by Kong et al. in 20 PCC/PGL patients regarding the role of combined PRRT and radiosensitizing chemotherapy [18], which included only 11 patients with PGL. Thus, we conducted a retrospective, single-arm, observational study to assess the additional benefit of concomitant ^177^Lu-DOTATATE and capecitabine therapy in PGL patients.

## Materials and methods

### Patients

This study involved patients with PGL treated between July 2009 and March 2017, at the Department of Nuclear Medicine, AIIMS, New Delhi, India.

### Patient inclusion and exclusion criteria

Eligibility for the ^177^Lu-DOTATATE therapy included the following: patients with incompletely resected or inoperable tumors, resistant to conventional therapies, disease progression despite first-line treatment options and negative scan findings on ^131^I-MIBG scintigraphy with a significant uptake pattern on ^68^Ga-DOTANOC PET/CT(positron emission tomography/computed tomography) scan (Krenning score > 3). Patients with baseline hemoglobin of less than 9.5 g/dL, platelet count less than 60,000/μL, total leukocyte counts less than 4000/μL, serum creatinine of greater than 1.4 mg%, serum bilirubin greater than 1.2 mg%, glomerular filtration rate (GFR) less than 60 mL/min/1.73 m^2^ BSA, Karnofsky Performance Status (KPS) < 40, Eastern Cooperative Oncology Group (ECOG) status > 3, and ^68^Ga-DOTANOC PET/CT scan showing Krenning score ≤ 2 were excluded from the study. All patients gave written informed consent prior to the therapy. Patients who had at least 2 consecutive cycles of PRRT with concomitant radiosensitizer capecitabine therapy were included for analysis.

### Treatment regimen and follow-up

As a routine, pre-therapy baseline diagnostic ^68^Ga-DOTANOC PET/CT scan was obtained in all patients. The PET/CT scans were acquired on a Biograph mCT scanner [Siemens] after 30–40 min of intravenous injection of 100–185 MBq ^68^Ga-DOTANOC. As a prerequisite, long-acting and short-acting somatostatin was stopped 4 to 5 weeks and 48 to 72 h, respectively, prior to ^177^Lu-DOTATATE therapy. Prior to every cycle of ^177^Lu-DOTATATE therapy, complete blood counts (CBC), kidney function tests (KFT), GFR, liver function tests (LFT), and tumor markers were documented in all patients.

^177^LuCl_3_ was procured from the Board of Radiation & Isotope Technology, Bhabha Atomic Research Centre, Mumbai, India, and DOTATATE peptide was obtained from ABX (GmBH, Radeberg, Germany). In-house radiolabeling of ^177^LuCl_3_ with DOTATATE was performed in our laboratory according to the method described by Das et al. [19]. The radiochemical purity of the labeled product was analyzed by thin-layer chromatography method, and only products with > 96% labeling efficiency were injected to patients.

Before starting amino acid infusion, appropriate measures were taken to prevent nausea and vomiting by administering anti-emetic (ondansetron) and/or corticosteroid (dexamethasone). The medications were repeated if necessary. A single-day kidney protection protocol was followed with a cocktail of lysine and arginine infused over 4 h, starting 30 to 60 min before the ^177^Lu-DOTATATE infusion. After 30 to 60 min from the start of amino acid infusion, 5.55 to 7.40 GBq (150–200 mCi) of ^177^Lu-DOTATATE was diluted in 50 mL of saline and infused over 30 min with a flow rate of 1.6 mL/min. Patients were monitored for 24 h after therapy for any acute adverse effects and were discharged when the radiation levels dropped below the permissible environmental level of radiation limits (50 μSv/h) at 1 m as per the National Regulatory Guidelines of Atomic Energy Regulatory Board (AERB). Appropriate radiation safety counseling was given to all patients before they were discharged from the hospital.

Based on our clinic protocol, a radiosensitizing oral chemotherapeutic drug, capecitabine, 1250 mg/m^2^ (Xeloda; Roche Products) was prescribed for 15 consecutive days (D0-D14) commencing on the morning of ^177^Lu-DOTATATE therapy. Capecitabine therapy was repeated at the time of each subsequent ^177^Lu-DOTATATE therapy. For each patient, post-therapy whole-body scan (WBS) was acquired 24–48 h after ^177^Lu-DOTATATE infusion on a dual head gamma camera (Symbia; Siemens). A parallel hole, low-energy high-resolution collimator with energy windows centered at 113 KeV and 208 KeV with 20% window width was used for the acquisition. Follow-up was performed at 2 weeks, 4 weeks, and 3 months after each cycle of ^177^Lu-DOTATATE therapy with CBC, KFT, GFR, and LFT to assess the toxicity according to the National Cancer Institute for Common Toxicity Criteria version 3.2.4 [20]. Patients on oral capecitabine were under observation for any adverse events due to the drug. Therapy was repeated at 12- to 16-week intervals in all patients except for those with disease progression despite ^177^Lu-DOTATATE therapy. Treatment was stopped in patients who completed 6 to 8 cycles of therapy with a maximum administered cumulative activity of 50 GBq or developed disease progression (PD) during treatment.

### Response evaluation

Biochemical response was assessed according to change in the trend of plasma chromogranin A (CgA) levels. Objective response was evaluated using the Response Evaluation Criteria in Solid Tumors (RECIST) 1.1 criteria [21]. KPS and analgesic score (AS) were used to assess the quality of life in all the patients. In patients with head and neck PGLs (H&N PGL), quality of life was assessed using EORTC QLQ-H&N35 questionnaire. The QLQ-H&N35 comprises 35 questions which incorporates 7 multi-item scales consisting of 24 questions that assess pain, swallowing, senses (taste and smell), speech, social eating, social contact, and sexuality and 11 single-item scales which are related to problems with teeth, opening mouth, dry mouth, sticky saliva, cough, feeling ill, painkillers, nutritional supplements, feeding tube, weight loss, and weight gain. All the answers to the questions were converted to a linear scale ranging from 0 to 100 as advocated by Fayers et al., King, and Bjordal et al. [22–24]*.* The values were reported as a mean and standard deviation. For all the questions, a higher value reflected the presence of intense symptoms and indicates more problems (i.e., higher score = poorer QoL). Toxicity was assessed according to the National Cancer Institute Terminology Criteria version 3.2.4 for adverse events. After 6 months of PRRT, the alterations in the dosages of anti-hypertensive drugs and symptoms were evaluated in patients on medication for secondary hypertension.

### Outcome endpoints

The primary endpoints of the present study were progression-free survival (defined as the time from initiation of ^177^Lu-DOTATATE-capecitabine therapy to documented disease progression) and overall survival (defined as the time from initiation of ^177^Lu-DOTATATE treatment-capecitabine therapy to death from any cause). Secondary endpoints included assessment of QoL in H&N PGLs, safety, and side-effect profile. Primary events were considered as progression of disease with an increase in the CgA levels and/or increase in the structural lesions as per RECIST 1.1 criteria or death, whichever occurred first.

### Statistical analysis

Continuous variables were calculated as mean, median, standard deviation (SD), standard error of mean (SEM), and range. The overall survival (OS) and progression-free survival (PFS) plots were constructed using the Kaplan-Meier method. *P* < 0.05 was considered as significant. Stata 11.2 (Stata Corp, College Station, Tex) was used for the analysis.

## Results

### Patient demographics

A summary of the demographic characteristics of patients is provided in Table 1. At our clinic, 29 patients were treated; however, 4 patients were excluded due to only one cycle of ^177^Lu-DOTATATE therapy administered at the time of analysis. Thus, the remaining 25 patients were included in the study for final analysis. The median follow-up duration using reverse K-M censoring method was 30 months (15–96 months) from the start of ^177^Lu-DOTATATE therapy. There were 19 male and 6 female patients with a mean age of 35.6 ± 11.5 years (14–65 years). In 5 (20%) patients, tumor involvement was confined to the primary site, and in the remaining 20 (80%) patients, lymph node and skeletal metastases were the most common metastatic sites (Table 1). Based on the locations of paragangliomas, sympathetic PGLs were found in 8 patients and parasympathetic PGLs in 17 patients. Seventeen patients underwent various prior treatments enumerated in Table 1. At the time of the commencement of treatment, (PRRT-capecitabine) 21 patients presented with documented disease progression. The patients received a mean total activity of 22.86 ± 9.54 GBq (range 14.43–50 GBq). The interval between each therapy cycle was 3 months with a median of 3 cycles (range 2–8 cycles).

**Table 1:** Demographic characteristics of patients

Characteristics	Value
Age (years) (mean ± SD; range)	35.6 ± 11.5 (14–65)
Gender	
Male	19 (76%)
Female	6 (24%)
Metastases	
None	5 (20%)
Lymph nodes	13 (52%)
Skeletal	13 (52%)
Liver	3 (12%)
Lungs	3 (12%)
Prior treatment	
Yes	19 (76%)
No	6 (24%)
Type of prior therapies	
Surgery	14 (56%)
Chemotherapy	5 (20%)
Radiotherapy	4 (16%)
^131^I-MIBG therapy	2 (8%)
Inoperable/no treatment affected	6 (24%)
Disease status at baseline	
Progressive disease	21 (84%)
Stable disease	4 (16%)
Anti-hypertensive drugs	14 (56%)
Number of ^177^Lu-DOTATATE therapy cycles (median, range)	3 (2–8)
Treatment duration	
Median (range)	30 (15–96)
Cumulative activity (GBq)	22.86 ± 9.54 (14.43–50)

### Morphological response

**Fig. 1:**
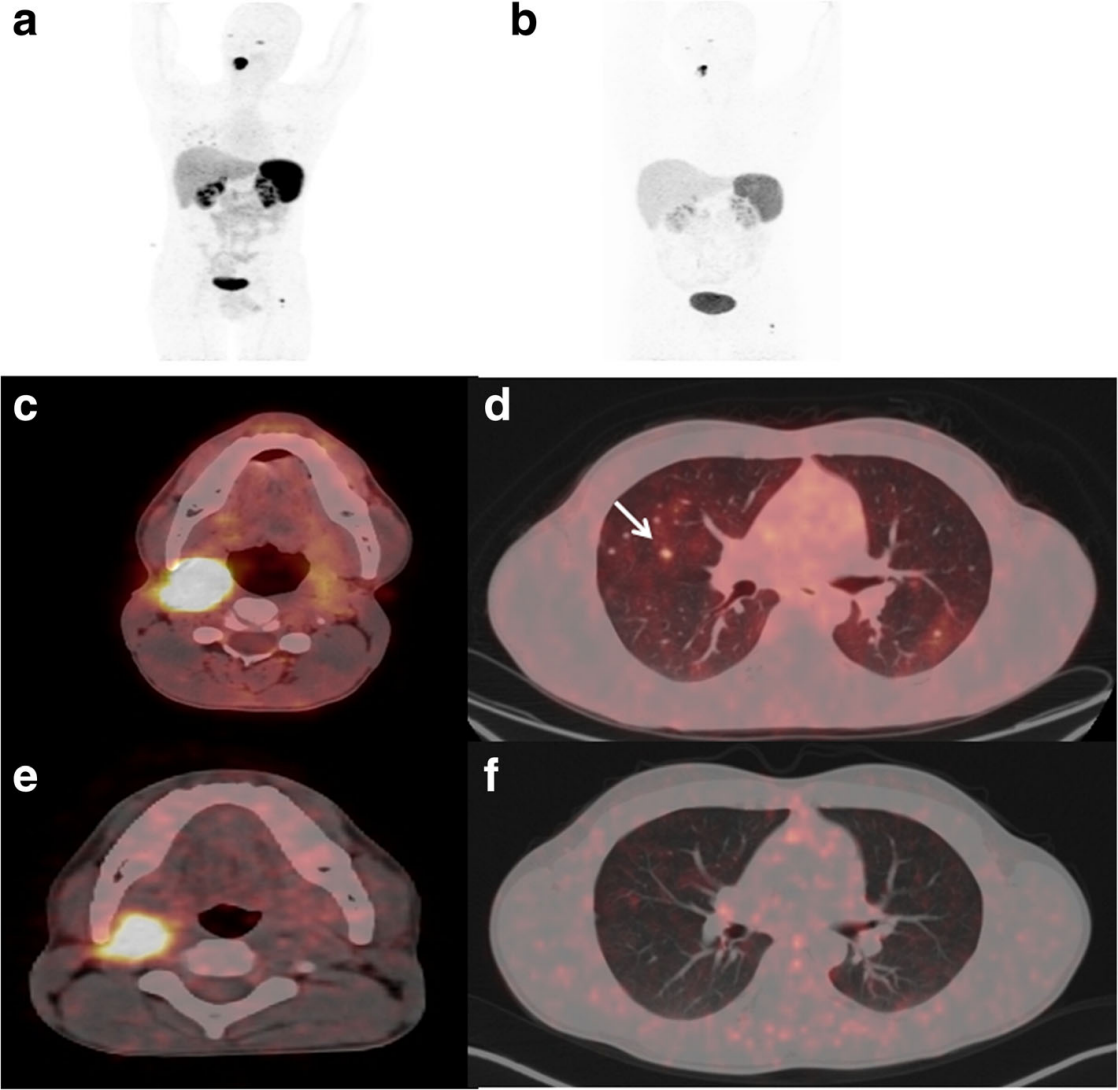
A 22-year-old male was diagnosed with inoperable metastatic right carotid body tumor in 2015. The baseline pre-therapy diagnostic ^68^Ga-DOTANOC PET/CT scan showed somatostatin receptor (SSTR) avid soft tissue mass lesion involving the right carotid body mass measuring 2.6 × 1.7 × 2.7 cm (**a**, **c**), with multiple B/L SSTR avid lung nodules (**d**) and skeletal metastases in the left femur (**a**). After 4 cycles of ^177^Lu-DOTATATE therapy, the follow-up diagnostic ^68^Ga-DOTANOC PET/CT scan showed partial morphological response with complete resolution of lung metastases and minimal residual disease in the primary tumor (**b**, **e**, **f**)

Table 2 summarizes the morphological response in the patient population according to RECIST 1.1 criteria. No patient achieved a complete response. Partial tumor response was observed in 7 patients (28%) (Fig. 1), stable disease in 14 patients (56%), and the remaining 4 patients (16%) had disease progression.

**Table 2:** Morphological, biochemical, and symptomatic response in patients

Response criteria	Response (*N*)
Morphological response (*N* = 25)	
Partial remission	7 (28%)
Stable disease	14 (56%)
Progressive disease	4 (16%)
Biochemical response (CgA) (*N* = 25)	
< 25% reduction	12 (48%)
25–50% reduction	4 (16%)
> 50% reduction	7 (28%)
Increase in CgA	2 (8%)
Symptomatic response (anti-hypertensive drugs) (*N* = 14)	
Reduction of anti-HTN drugs	6 (43%)
No change	8 (58%)

### Biochemical response post-PRRT

Among the 25 patients, CgA was available in 24 patients. Interestingly, 21 of the 24 patients showed a decrease in the CgA level; however, only 7 patients showed a significant decrease in the CgA levels > 50% (Table 2).

### Survival

**Fig. 2:**
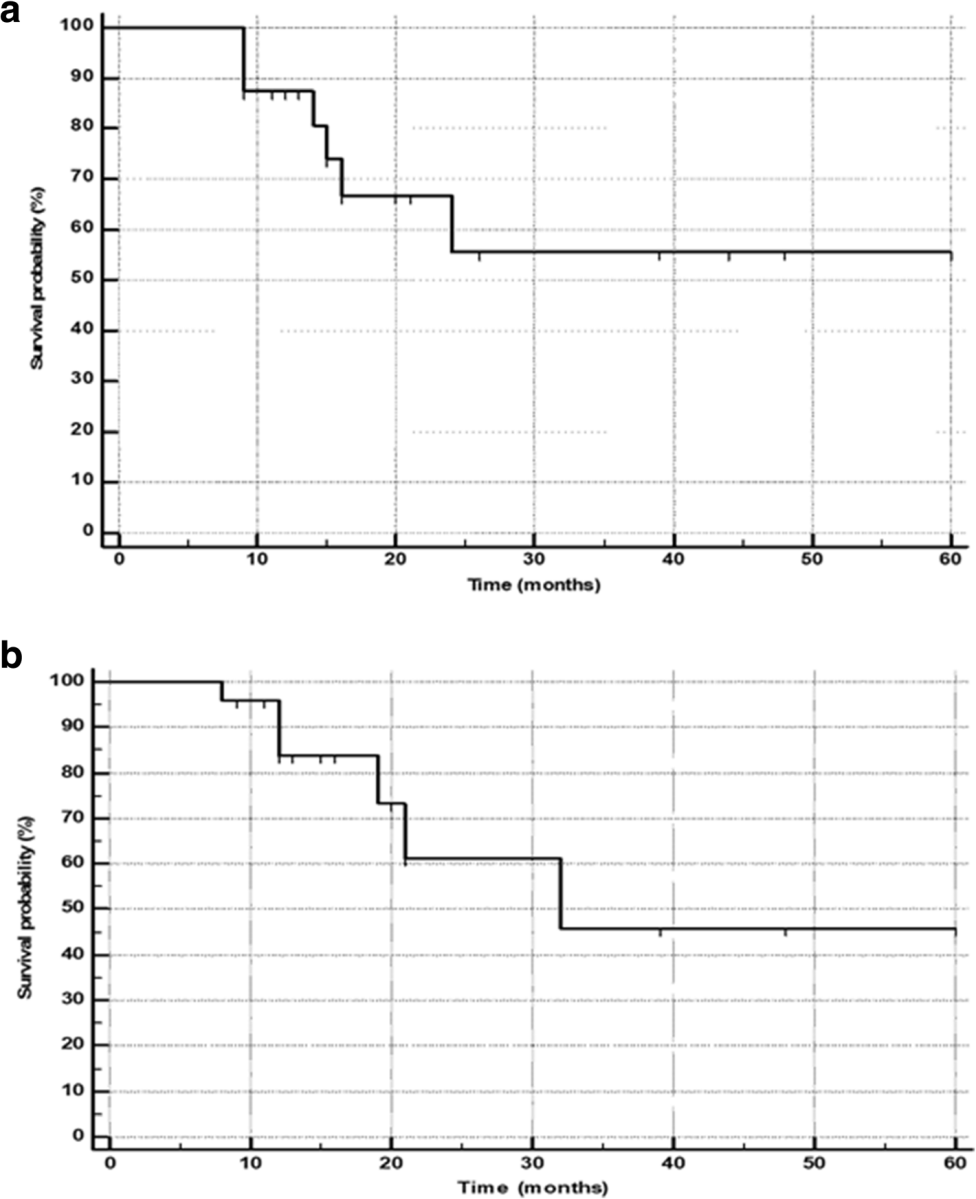
**a** Kaplan-Meier plots: overall survival function. **b** Kaplan-Meier plots: progression-free survival

**Fig. 3:**
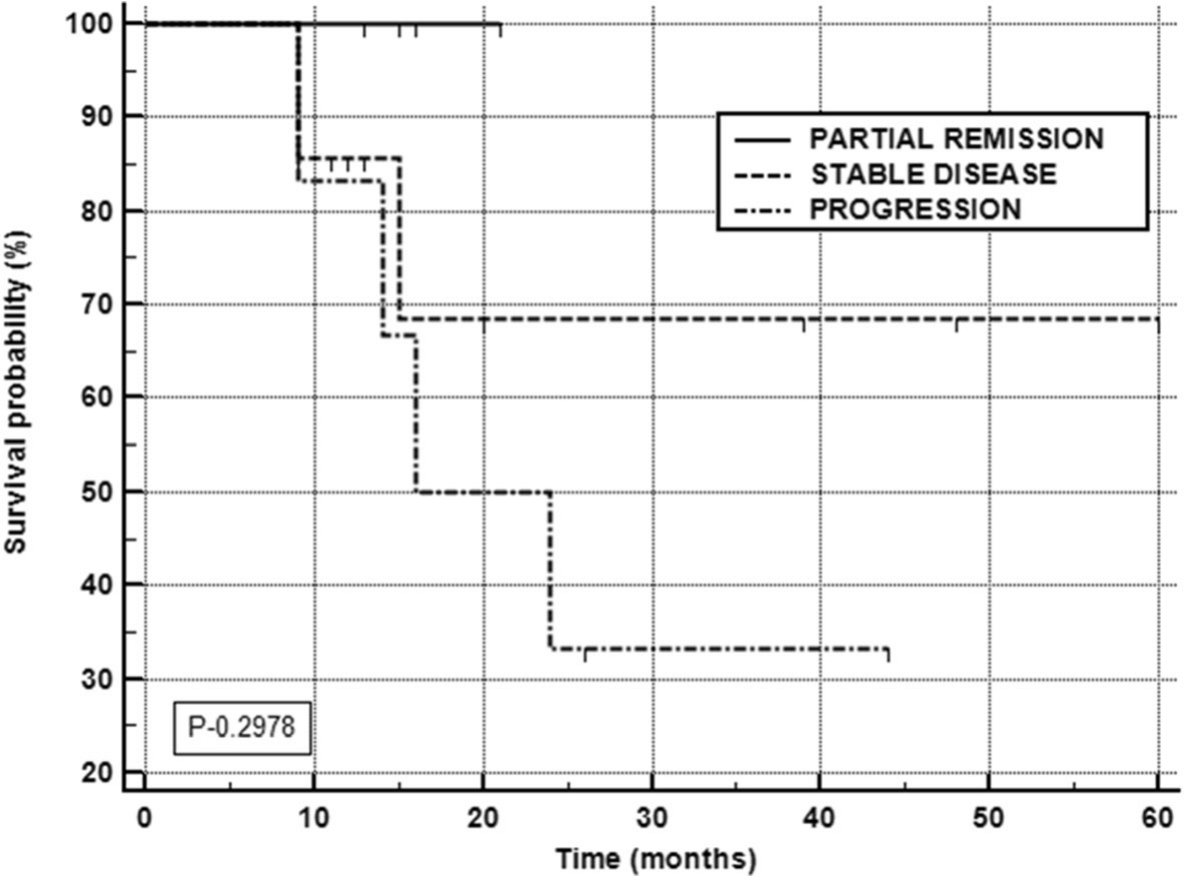
Overall survival according to morphological response (RECIST 1.1 criteria)

At the time of the final analysis, 18 patients were alive and the remaining 7 (28%) patients died of disease. The median overall survival in the total population was not reached (Fig. 2a). The median PFS predicted based on RECIST 1.1 criteria was 32 months (Fig. 2b). The median OS in patients with progressive disease was 16 months (95% CI 14–24 months), while it was not attained in the partial response and stable disease groups (Fig. 3). On subgroup analysis, as expected, a significant reduction in the median OS was observed in PD group patients compared to the other groups (*P* < 0.0001) (Fig. 3).

### Quality of life assessment (QoL)

In the group with partial tumor regression, the KPS showed nearly significant improvement from 62.5 ± 17 to 80 ± 8 (*P*, 0.068). However, interestingly, patients with stable disease also showed a significant improvement in the KPS from 66.9 ± 10.4 to 78.5 ± 9.4 (*P* < 0.002). A strikingly significant decrease in the KPS score was observed before and after therapy in the progressive disease group patients (*P* < 0.008). The analgesic score showed a corresponding decrease from 4.3 ± 1.2 to 3.1 ± 1.1 (*P* < 0.000) (Table 3).

**Table 3:** KPS and analgesic scores pre- and post-PRRT

KPS	Baseline	Post-therapy	*P* value
KPS (in PR patients)	62.5 ± 17	80 ± 8	0.068
KPS (in SD patients)	66.9 ± 10.4	78.5 ± 9.4	0.002
KPS (in PD patients)	65 ± 8.3	18 ± 29.9	0.008
Analgesic score	4.3 ± 1.2	3.1 ± 1.1	0.000

Seventeen of 25 (68%) patients had H&N PGLs. The detailed QLQ-H&N35 reports at two time points for these set of patients are compared and enumerated in Tables 4 and 5. Among the symptom scales, pain, difficulty in swallowing, opening mouth, coughing, and feeling ill were high at baseline and decreased drastically post ^177^Lu-DOTATATE therapy. A significant reduction in the intake of painkillers and nutritional supplements was observed. While a significant improvement in the social eating scale was also noted, a marginally significant improvement of social contact at the time of assessment was observed. An improvement in all other symptoms was observed, but the differences were insignificant.

**Table 4:** EORTC QLQ-H&N35 scoring at the onset and end of assessment in 17 H&N PGL patients

Variables	Baseline QLQ	Post-therapy QLQ	*P* value
Pain	51.8 ± 22.7	26.8 ± 20.8	0.001
Swallowing	31.4 ± 33.3	17.31 ± 16.6	0.001
Senses (taste and smell)	11.1 ± 24.9	10.1 ± 24.3	0.903
Speech	34.5 ± 22.2	30.2 ± 22.2	0.296
Social eating	31.1 ± 33.3	16.6 ± 16.6	0.002
Social contact	29.6 ± 23.7	19.6 ± 19.5	0.074
Sexuality	28.7 ± 25	31.4 ± 16.6	0.562
Teeth	25.9 ± 33.3	16.6 ± 0.1	0.244
Opening mouth	44.4 ± 50	24 ± 33.3	0.158
Dry mouth	18.5 ± 33.3	11.1 ± 0.1	0.125
Sticky saliva	11.1 ± 16	3.7 ± 10.7	0.112
Coughing	38.8 ± 33.3	20.3 ± 16.6	0.042
Felt ill	48.1 ± 26.1	27.7 ± 32.8	0.046
Painkiller	83.3 ± 38.3	18.5 ± 17	0.000
Nutritional supplement	44.4 ± 51.5	18.6 ± 12.7	0.046

**Table 5:** EORTC QLQ-H&N35 scoring in the morphologically stable head and neck paraganglioma assessed at the onset and end of the assessment

Variables	Baseline QLQ	Post-therapy QLQ	*P* value
Pain	51.8 ± 22.7	26.8 ± 20.8	0.001
Swallowing	31.4 ± 33.3	17.31 ± 16.6	0.786
Senses (taste and smell)	11.1 ± 24.9	10.1 ± 24.3	0.903
Speech	34.5 ± 22.2	30.2 ± 22.2	0.312
Social eating	31.1 ± 33.3	16.6 ± 16.6	0.107
Social contact	29.6 ± 23.7	19.6 ± 19.5	0.175
Sexuality	28.7 ± 25	31.4 ± 16.6	0.705
Teeth	25.9 ± 33.3	16.6 ± 0.1	0.244
Opening mouth	51.2 ± 50	25.6 ± 33.3	0.158
Dry mouth	17.9 ± 17.2	10.25 ± 16	0.251
Sticky saliva	11.1 ± 16	3.7 ± 10.7	0.112
Coughing	38.4 ± 18.4	20.5 ± 21.6	0.002
Felt ill	43.5 ± 33.3	25.6 ± 33.3	0.031
Painkiller	83.3 ± 38.3	18.5 ± 17	< 0.0001
Nutritional supplement	38.4 ± 50.6	5.1 ± 12.1	0.062

The secondary hypertension was noted in 14/25 (56%) patients. At 6 months post-PRRT therapy, anti-hypertensive medications were reduced in 6/14 (43%) patients, 8 patients were maintained on the same dosage as the baseline, and none of the patients were completely off anti-hypertensive medications (Table 2).

### Toxicity

Treatment-related adverse events during the ^177^Lu-DOTATATE therapy were recorded in all patients. Eight percent of the patients experienced vomiting, and 16% experienced nausea during amino acid infusion. Blood pressure fluctuations were observed in 2 patients. No early or delayed tumor lysis syndrome was observed in any patient.

Various blood parameters were recorded at baseline and post-therapy. At baseline, 4 patients had grade I anemia and remained in grade I hematotoxicity all through the treatment. Three patients developed grade I lymphopenia. None of the patients developed thrombocytopenia or leukopenia. There was no significant difference in the hemoglobin levels (*P*, 0.477) and platelet counts (*P*, 0.5474) after therapy. Regarding the renal parameters, there was no significant difference observed in blood urea, serum creatinine, and GRF levels between the pre-therapy and post-therapy (Table 6). Two patients encountered a decrease in the GFR levels below 60 mL/min/1.73 m^2^ BSA with a corresponding elevation in the creatinine levels. None of the patients experienced hepatotoxicity. All patients were prescribed concomitant capecitabine therapy, and no side effects were observed in any patient.

**Table 6:** Comparison of pre- and post-therapy laboratory parameters

Laboratory parameters	Baseline	Post-therapy	*P* value
Hematotoxicity			
Hemoglobin, g/dL	12.9 ± 1.5	12.5 ± 1.5	0.477
Platelets, 100,000/mm^3^	278.3 ± 92.2	256 ± 85.7	0.547
Leukocytes, cells/mm^3^	7749 ± 2121	7435 ± 2561	0.223
Nephrotoxicity			
Blood urea, mg/dL	20.7 ± 7.4	23.35 ± 11.4	0.407
Serum creatinine, mg/dL	0.84 ± 0.2	0.82 ± 0.2	0.605
GFR, mL/min/1.73 m^2^ BSA	93.8 ± 25.2	92.7 ± 24.8	0.984
Hepatotoxicity			
Serum bilirubin, mg/dL	0.57 ± 0.13	0.51 ± 0.45	0.442

All parameters are explained as mean ± SD

## Discussion

The role of PRRT in GEP NETs is well established with an enormous availability of literature with a recent study published by Strosberg et al. who have demonstrated good prognosis [25]. However, the data concerning the outcome of PRRT in advanced PGLs is scarce with very few retrospective studies reported [18, 26–28]. Moreover, there is only a single study by Kong et al. reporting the synergistic effect of PRRT and various chemotherapeutic drugs and is confined to 9 out of 20 patients with PCC/PGL [18]. Our recent publication demonstrated the beneficial effect of combined capecitabine and ^177^Lu-DOTATATE in GEP-NET patients [17]. On this basis, we were keen to observe the role of synergistic approach in PGL patients and its effect on the OS and PFS.

Among the radionuclide therapies, ^131^I-MIBG therapy is considered as the standard of care. Recently (July 2018), the US Food and Drug Administration has approved the drug Adzera (MIBG-I131) in patients 12 years or older to treat PCC and PGL tumors that are surgically unresectable and have metastasized based on the results of a clinical trial in 68 patients. A systemic review and meta-analysis by Van Hulsteijn et al. [11] included 94 PGL patients treated with ^131^I-MIBG therapy and observed that a slightly higher proportion of patients with PGL had achieved objective response compared to PCC patients (0.04: CR and 0.30: PR for patients with PGL vs 0.01: CR and 0.28: PR for patients with PCC). Few other studies have observed objective response rates varying from 36 to 57% of patients with PCC/PGL [8–10]. However, hematological toxicities and long hospitalizations were the main pitfalls of ^131^I-MIBG therapy. Hematological toxicities comprised the most common side effects of ^131^I-MIBG therapy and were observed in 26% of the patients by Gedik et al. [9]. In agreement with the above studies, Gonias et al. [10] also observed grade 3/4 neutropenia in 87% and thrombocytopenia in 83% of patients.

Thus, the eligibility criteria for ^131^I-MIBG therapy depend on various factors such as hematological profile, clinical status, and the degree of avidity on ^123^I/^131^I-MIBG whole-body scintigraphy. Based on the above discussion, one single treatment protocol does not fit all.

To the best of our knowledge, prospective RCTs involving head-to-head comparison of ^177^Lu-DOTATATE with ^131^I-MIBG have not been published. However, in this regard, Nastos et al. [29] attempted to retrospectively compare the therapeutic effect of various systemic radionuclide therapies such as ^131^I-MIBG, ^90^Y-DOTATATE, or ^177^Lu-DOTATATE in 22 patients with progressive/metastatic PGLs and PCCs (*N* = 15 PGLs). Among all the therapies, PRRT treatment offered the best response and improved OS, PFS, and event-free survival (EFS) compared to other systemic therapies. There is a high potential that PRRT frequently be used to treat PGL/PCC in the future. A single-arm, ongoing clinical trial (NCT03206060) on ^177^Lu-DOTATATE (Lutathera) therapy in inoperable PCC/PGL is in progress and is expected to complete in 2024. Kong et al. evaluated the efficacy of concomitant PRRT and chemotherapy in 20 patients with PCC/PGL of whom 11 patients had PGL. Though the response was not specifically mentioned for PGL, overall they observed 29% patients with PRRT induced morphological disease regression (no CR) [18] that is similar to the results of our study (OR 28%). However, van Essen et al. [26] have shown slightly higher ORR of 33%. Unlike our study, the above authors have considered minimal response also as objective response and have incorporated PCC patients. Therefore, it is not surprising that they have attained marginally higher remission rates compared to our results. Interestingly, Forrer et al. [16] in a similar number of patients have demonstrated an ORR of 28%. On an extensive literature search, 5 studies have previously reported response rates that range from 11 to 28% with ^177^Lu-DOTATATE monotherapy [26–28] (Table 7).

**Table 7:** Comparison of various reports on the efficacy of PRRT in PGL/PCC patients

Author and year	Patient number	PR	SD	PD	Death	Follow-up (median, months)	Median OS (months)	Median PFS (months)	TTP
van Essen et al. 2006 [27]	12	2 (17%)	6 (50%)	4 (33%)	2 (16%)	13 (4–30)	–	–	–
Forrer et al. 2008 [16]	25	2PR + 5MR (28%)	13 (52%) Mixed response 2 (8%)	6 (24%)	–	*19 (6–50)	–	–	3 to > 42 Mean not reached
Zovato et al. 2012 [28]	4	2 (50%)	2 (50%)	0	0	*15.8 (12–25)	–	–	–
Pinato et al. 2016 [29]	5	1 (20%)	3 (60%)	1 (20%)	2 (40%)	13	–	17 (0–78)	Not reached
Kong et al. 2017 [18]	20	CT scan			5 (25%)	28	Not reached	39	–
		29% SSTR	50%	14%					
		47%	41%	12%					
Present study	25	5 (20%)	16 (64%)	4 (16%)	7 (28%)	30 (9–60)	Not reached	32	13

*PR* partial response, *SD* stable disease, *PD* progressive disease, *OS* overall survival, *PFS* progression-free survival, *TTP* time to progression, *MR* minimal response, *CT scan* computed tomography scan, *SSTR imaging* somatostatin receptor imaging

*Mean follow-up duration in months

The median PFS in our patient population was in consonance with the results of Kong et al. (32 months vs 39 months). We also observed a similar disease progression percentage (16%, 4/25) compared to Kong et al. [18] (12%, 2/17). The median OS for the patient population was not attained because less than 50% of deaths had occurred and was consistent with the results of Kong et al. [18]. The inherent indolent nature of the tumor may be the reason for markedly long overall survival. When sub-stratified according to the RECIST 1.1 criteria, patients with progressive disease had a median OS of 16 months, while it was not reached in the other categories of patients.

The quality-of-life assessment was conducted at baseline and after 3 months of the last cycle administered to the patient. Most of the patients reported symptomatic relief and improved general well-being after the therapy. Significant improvement in all the scales was observed during the ^177^Lu-DOTATATE therapy. When sub-stratified according to the disease status during the follow-up, patients with PD experienced worsening of symptoms and a corresponding decrease in the QoL compared to the baseline status. Appreciable improvement is the social contact and social eating scales were observed before and after therapy. Despite stable disease on morphological imaging, a significant improvement on the pain scale was noted in the QoL after the therapy (Table 5). In this context, ^177^Lu-DOTATATE therapy plays a crucial role in patients with inoperable/unresectable tumors. In this scenario, ^177^Lu-DOTATATE therapy can be used as an adjuvant to surgery. Secondary HTN was effectively controlled in a majority of the patients with relief in symptoms and improved the quality of life. Similar findings were confirmed by Kong et al. [18].

All the hematological toxicities were transient. At the time of recruitment, 4 patients had creatinine levels > 1 but < 1.3 mg/dL, but the GFR was normal. During the treatment course, 2 patients experienced a decrease in GFR levels below 60 mL/min/1.73 m^2^ BSA, but the decrease was minimal. The low toxicity of ^177^Lu-DOTATATE therapy makes it favorable over ^131^I-MIBG therapy.

However, few limitations in the present study are recognized. Firstly, the numbers of patients in the study are limited, but this is the largest among the series exclusively reporting the therapeutic outcome of PGL patients with synergistic ^177^Lu-DOTATATE and capecitabine therapy. Secondly, this is only a retrospective, single-arm, single-institutional study where patients were administered uniform concomitant treatment. Unfortunately, we could not compare our results with a historic control group from previously published literature as the sample size reported are small; hence, our results should be interpreted with caution. To overcome this limitation, we recommend two-armed multi-institutional randomized control trials to compare the synergetic approach of ^177^Lu-DOTATATE and capecitabine therapy versus ^177^Lu-DOTATATE monotherapy in PGLs.

Thirdly, catecholamines and metanephrines were not available in all patients; hence, the data could not be presented.

## Conclusion

In view of the small sample size, we could not prove the superiority of concomitant ^177^Lu-DOATATATE-capecitabine therapy. However, this preliminary study showed that concomitant therapy in advanced PGL is not inferior to the published data of ^177^Lu-DOTATATE monotherapy and warrants a large randomized control trial to validate the beneficial effect of synergistic radiosensitizer capecitabine therapy with ^177^Lu-DOTATATE PRRT in PGL.

## Abbreviations

AERB: Atomic Energy Regulatory Board
AS: Analgesic score
CBC: Complete blood counts
CgA: Chromogranin A
CR: Complete remission
ECOG: Eastern Cooperative Oncology Group
GEP NETs: Gastroenteropancreatic neuroendocrine tumors
GFR: Glomerular filtration rate
H&N: Head and neck
KFT: Kidney function tests
KPS: Karnofsky Performance Status
LFT: Liver function tests
LuCl_3−_: Lutetium chloride
MIBG: Metaiodobenzylguanidine
MR: Minimal response
NET: Neuroendocrine tumors
OS: Overall survival
PCC: Pheochromocytoma
PD: Progression disease
PET/CT: Positron emission tomography/computed tomography
PFS: Progression-free survival
PGL: Paraganglioma
PR: Partial remission
PRRT: Peptide receptor radionuclide therapy
QoL: Quality of life assessment
RECIST: Response Evaluation Criteria in Solid Tumors
SEM: Standard error of mean
SSTR: Somatostatin receptors
TTP: Time to progression
WBS: Whole-body scan
